# Arbuscular Mycorrhizal Fungi Inoculation and Different Phosphorus Fertilizer Levels Modulate Phosphorus Acquisition and Utilization Efficiency of Alfalfa (*Medicago sativa* L.) in Saline-Alkali Soil

**DOI:** 10.3390/plants15010114

**Published:** 2025-12-31

**Authors:** Shangzhi Zhong, Pengxin Hou, Mingliu Yu, Wei Cao, Xiangjian Tu, Xiaotong Ma, Fuhong Miao, Qibo Tao, Juan Sun, Wenke Jia

**Affiliations:** Shandong Key Laboratory for Germplasm Innovation of Saline-alkaline Tolerant Grasses and Trees, College of Grassland Science, Qingdao Agricultural University, Qingdao 266109, China; zhongsz@qau.edu.cn (S.Z.); hpx1031@163.com (P.H.); yumingliu0929@163.com (M.Y.); vv20200115@163.com (W.C.); tuxiangjian1998@163.com (X.T.); maxiaotong0201111@163.com (X.M.); miaofh@qau.edu.cn (F.M.); taoqibo@qau.edu.cn (Q.T.)

**Keywords:** P uptake strategies, mycorrhizal contribution, root characteristics, rhizosphere carboxylates, microbial biomass

## Abstract

Phosphorus (P) is a key nutrient limiting crop growth and productivity, particularly in saline-alkali soils with low P availability. Arbuscular mycorrhizal fungi (AMF) have the potential to enhance P uptake in alfalfa (*Medicago sativa* L.); however, the synergistic effects and underlying biological mechanisms by which AMF improve P acquisition and utilization efficiency under varying P application levels remain unclear. To explore P acquisition strategies associated with AMF status, root morphology traits, rhizosphere carboxylate exudation, soil properties and microbial biomass, we conducted a pot experiment growing alfalfa in saline-alkali soil under four P application levels (0, 5, 10, and 20 mg kg^−1^), with or without AMF inoculation. Our results showed that AMF colonization and P application synergistically increased alfalfa biomass and shoot/root P concentrations. Notably, at a low P application level of 5 mg kg^−1^, the mycorrhizal contribution to P absorption and P-utilization efficiency reached their highest levels, while both declined under high P conditions (20 mg kg^−1^), suggesting an interaction between P availability and AMF efficacy. Structural equation modeling (SEM) and regression analysis revealed that rhizosphere carboxylate concentrations were positively associated with P-utilization efficiency, whereas soil available P, microbial biomass P (MBP) and carbon (MBC) negatively affected it. Among these factors, AMF-induced enhancement of rhizosphere carboxylate exudation played a critical role in promoting P-utilization efficiency in alfalfa under low-P conditions. In contrast, higher P availability reduced rhizosphere carboxylate concentrations, resulting in lower P-utilization efficiency. In conclusion, the combination of AMF colonization and low P application synergistically improves P acquisition and utilization efficiency in alfalfa, providing valuable insights for sustainable nutrient management in saline-alkali soils with limited P availability.

## 1. Introduction

Phosphorus (P) is an essential macronutrient for plant development, playing a pivotal role in numerous physiological and biochemical processes, including energy transfer, nucleic acid synthesis, membrane formation, and signal transduction [1,2,3,4]. Despite its importance, the availability of plant-accessible P in many agricultural soils is extremely limited, as most soil P exists in the form of insoluble organic or mineral complexes with high adsorption and low mobility [5,6]. This constraint is particularly pronounced in saline-alkali soils, where high pH, elevated ion concentrations, and poor soil structure further exacerbate P fixation and reduce plant P uptake efficiency [7]. Simultaneously, low P availability impairs root growth, thereby increasing plants’ susceptibility to Na^+^ toxicity [8,9]. Consequently, P deficiency is often a primary limiting factor for crop growth and productivity in such environments, leading to reduced biomass accumulation, delayed flowering, low fruit set, and yield reduction [6,10,11,12]. To overcome this limitation, P fertilizers have been widely applied to agricultural systems to replenish available P pools and support crop production [13]. However, the sustainability of P fertilizer application has attracted growing scrutiny. Beyond the non-renewable phosphate rock—the primary source of P fertilizers—low P utilization efficiency in agricultural systems further drives the excessive use of such fertilizers [14]. A large portion of applied P is often not readily absorbed by crops; instead, it is adsorbed to or precipitated with soil solid phases, thereby reducing its bioavailability for subsequent crop uptake [15,16]. This low P utilization efficiency consequently increases economic costs for farmers, as additional P inputs are required to meet crop nutrient requirements. Furthermore, such excessive application leads to substantial environmental degradation, including P-rich nutrient runoff into water bodies, subsequent eutrophication, and biodiversity loss in aquatic ecosystems [17]. These challenges underscore the urgent need to develop more efficient and sustainable strategies for improving P acquisition and utilization efficiency, especially in low-P availability saline-alkali soils where conventional fertilization practices are less effective [18].

Among various biological strategies to enhance plant P acquisition, the symbiotic association between arbuscular mycorrhizal fungi (AMF) and plant roots represents one of the most effective and ecologically sound solutions [5,19,20]. AMF, belonging to the phylum of *Glomeromycota*, form a mutualistic relationship with approximately 80% of terrestrial plant species, which can promote plant growth by promoting nutrient uptake and carbohydrate utilization [13,21]. Through an extensive network of extraradical hyphae that extend far beyond the depletion zone of plant roots, AMF significantly increase the absorptive surface area available for nutrient uptake, particularly immobile nutrients such as P [22,23,24,25]. These fungi enhance nutrient uptake, particularly under environments with low available P, by extending the root system through a network of fungal hyphae [26,27]. The hyphae penetrate the root epidermis to colonize cortical cells and form arbuscules, composed of fungal hyphae ensheathed in a modified form of the cortical cell plasma membrane termed the periarbuscular membrane (PAM) [28,29]. This symbiotic relationship is especially beneficial in phosphorus-deficient soils, as AMF can mobilize P that would otherwise be unavailable to plants [4,26,30]. AMF can also stimulate the activity of acid phosphatases and increase the exudation of carboxylates-organic anions such as citrate and oxalate-which can chelate metal cations like Ca^2+^, Fe^3+^, and Al^3+^ that otherwise bind P into insoluble complexes [31,32]. This chelation enhances P solubility and bioavailability in the rhizosphere, especially in calcareous or saline-alkali soils where P is often immobilized [33,34]. In addition, carboxylates may contribute to localized acidification of the rhizosphere, further promoting the desorption and mobilization of both organic and inorganic P fractions [5,8]. In addition to enhancing P acquisition, AMF improve plant resilience under various abiotic stresses, including drought, salinity, and nutrient imbalances [22,23,27]. In saline-alkali soils, AMF can improve rhizosphere conditions by modulating ion homeostasis, enhancing osmotic adjustment, and promoting antioxidant activity, thereby mitigating the negative impacts of salt-induced physiological stress on plants [35,36]. Furthermore, AMF colonization can alter root morphology and activate P transporter genes, facilitating more efficient internal P utilization [8,13,37]. The effectiveness of AMF, however, can vary depending on the fungal species, host plant genotype, soil properties and environmental conditions [11,36,38], suggesting the need for context-specific evaluations of AMF performance. Understanding the functional roles of AMF in saline-alkali soils under phosphorus-deficient conditions is therefore critical for developing low-input, sustainable strategies to improve nutrient use efficiency in agricultural systems.

Despite extensive studies on the role of AMF in enhancing plant P uptake and alleviating abiotic stresses, relatively few investigations have focused on their interactions with alfalfa under simultaneous saline-alkali and low-P stress conditions. Alfalfa (*Medicago sativa* L.), as a perennial legume with high forage value, is frequently cultivated in marginal lands where soil salinization and P deficiency co-occur, severely limiting its productivity and quality [28,38]. While a number of studies have demonstrated that AMF can enhance alfalfa growth and nutrient acquisition in normal conditions or single-stress [22,23], the mechanisms by which AMF modulate P acquisition and utilization efficiency under combined saline-alkali and low-P stress remain poorly understood. In particular, due to technical challenges and the complexity of studying multiple stress factors simultaneously, there is a lack of systematic research integrating physiological, biochemical, and molecular indicators to elucidate the AMF-mediated enhancement of P use efficiency in alfalfa under such dual-stress environments. Therefore, the present study aims to investigate the effects of AMF inoculation on phosphorus acquisition, distribution, and utilization efficiency in *M. sativa* grown in saline-alkaline soil under low-P conditions. Two primary hypotheses were investigated in this study: (1) AMF colonization and low-level P application could synergistically increase the growth and yields of alfalfa in the saline-alkali soil; (2) AMF symbiosis could improve soil environment conditions and root functional traits, especially the carboxylates in root exudates, thereby improving the efficiencies of P acquisition and utilization in alfalfa under low-P supply conditions. The findings will provide theoretical and practical support for the application of AMF in improving legume forage production and soil health in degraded ecosystems.

## 2. Results

### 2.1. AMF Status and Root Morphology Characteristics

In our experiment, no mycorrhizal structures were observed in the non-inoculated (−AMF) of alfalfa seedlings under four P application conditions (Table 1). In the pots subjected to +AMF treatment, AMF successfully colonized the alfalfa roots; meanwhile, the different concentrations of P application treatments significantly affected the AMF colonization rate, spore density and hyphal length (*p* < 0.01, Table 1). The AMF colonization rate, spore density, and hyphal length reached the highest values in the P5 treatment, whereas these parameters reached the lowest values in the P20 treatment (Table 1). Both AMF inoculation and P application treatments had significant effects on root morphology characteristics, including total root length, root diameter, root surface area and specific root length (*p* < 0.05, Table 2). As P application level increased total root length, root diameter and root surface area progressively increased; meanwhile, total root length and root surface area increased in +AMF pots compared to −AMF pots within the P0 and P5 treatments, but decreased within the P20 treatment (Table 2). Notably, specific root length exhibited a decreasing trend with increased P application concentration, and tended to be higher in +AMF treatments, but no significant difference was observed between −AMF and +AMF treatments (*p* > 0.05).

### 2.2. Plant Biomass and P Content, P Utilization and Acquisition Efficiency

**Figure 1:**
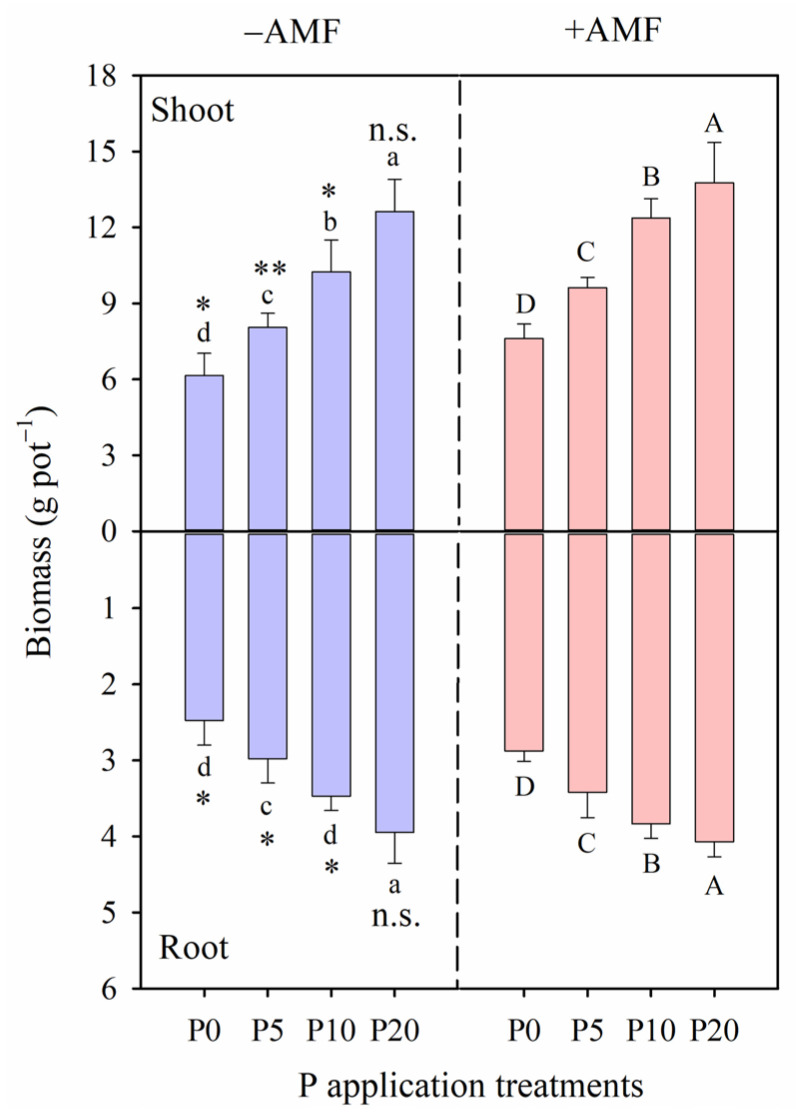
Shoot and root biomass of alfalfa growing in without arbuscular mycorrhizal fungi inoculation (−AMF) and with AMF inoculation (+AMF) under different phosphorus (P) application rates (P0: 0 mg kg^−1^, P5: 5 mg kg^−1^, P10: 10 mg kg^−1^, and P20: 20 mg kg^−1^). The same treatments below. Different lowercase letters and capital letters indicate significant differences among the P application rates under the −AMF and +AMF treatment, respectively. Levels of significance for differences between −AMF and +AMF treatments, which are indicated as: n.s. = not significant, * = *p* < 0.05, ** = *p* < 0.01. Vertical dashed lines denote the separation of −AMF and +AMF treatment. Data are reported as arithmetic mean ± standard error (n = 5).

Both AMF inoculation and P application treatments increased shoot biomass, root biomass, and total biomass, except for the root/shoot ratio (Table 2 and Table 3). Notably, under the +AMF treatment, shoot, root and total biomass were significantly higher versus the −AMF treatment, except for root biomass in the P20 treatment, where no significant difference was observed (*p* > 0.05, Figure 1). Relative to the −AMF and P0 (control) treatment, the increase in total biomass for the +AMF plants compared to the −AMF plants were 52% (+AMF) vs. 28% (−AMF) in the P5 treatment, 89% (+AMF) vs. 59% (−AMF) in the P10 treatment, and 107% (+AMF) vs. 92% (−AMF) in the P20 treatment (Table 2).

**Figure 2:**
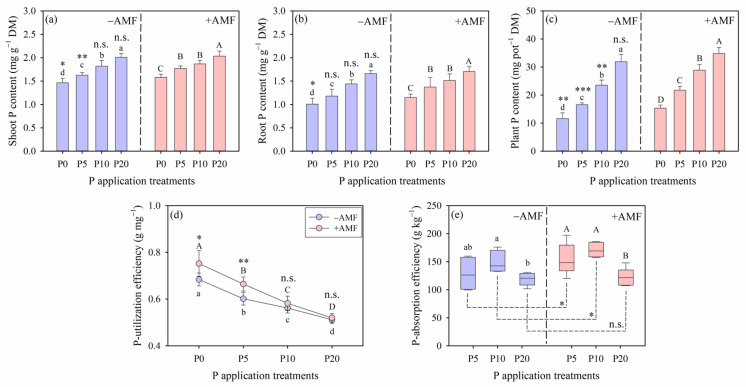
Shoot phosphorus (P) content (**a**), root P content (**b**), plant P content (**c**), P-utilization efficiency (**d**) and P-acquisition efficiency (**e**) of alfalfa plants growing without (−AMF) and with (+AMF) arbuscular mycorrhizal fungi inoculation under different P application treatments. Different lowercase (for −AMF) and capital (for +AMF) letters indicate significant differences among P application rates. Levels of significance for differences between −AMF and +AMF treatments, which are indicated as: n.s. = not significant, * = *p* < 0.05, ** = *p* < 0.01, *** = *p* < 0.001. Vertical dashed lines denote the separation of −AMF and +AMF treatment. Data are reported as arithmetic mean ± standard error (n = 5).

The AMF inoculation and P application treatments significantly influenced plant P concentrations (including shoot, root and total plant P), P-utilization efficiency and P-acquisition efficiency. However, a significant interaction between AMF inoculation and P application was observed only for P-utilization efficiency (*p* < 0.05; Table 3). For both +AMF and −AMF alfalfa plants, shoot and root P concentrations increased with higher P application levels, reaching their maximum values in the P20 treatment (Figure 2). Although AMF inoculation enhanced shoot and root P concentrations, there were no significant differences in the total P content between +AMF and −AMF plants (*p* > 0.05, Figure 2a–c). The relative increase in total P content due to AMF inoculation, compared to −AMF plants, was 33% for the P0 treatment, 31% for the P5 treatment, 22% for the P10 treatment, and 9% for the P20 treatment (Figure 2c). Notably, P application treatments significantly influenced the mycorrhizal P absorption contribution, which reached the highest and lowest values in the P5 and P20 treatments, respectively (Table 1). Compared to the P0 treatment, the P20 treatment decreased the mycorrhizal contribution by 59%.

For both −AMF and +AMF treatments, P-utilization efficiency gradually declined with increasing P application levels (Figure 2d). AMF inoculation significantly enhanced P-utilization efficiency in the alfalfa plants, with significant differences in the P0 and P5 treatments (*p* < 0.05). P-acquisition efficiency increased with higher P application levels, peaked at the P10 treatment, and then decreased, showing the lowest efficiency under both +AMF and −AMF treatments at the P20 treatment (Figure 2e). AMF inoculation significantly improved P-acquisition efficiency in the alfalfa plants at the P5 and P10 treatments (*p* < 0.05), but not in the P20 treatment (*p* > 0.05, Figure 2e).

### 2.3. Rhizosphere Carboxylates

**Figure 3:**
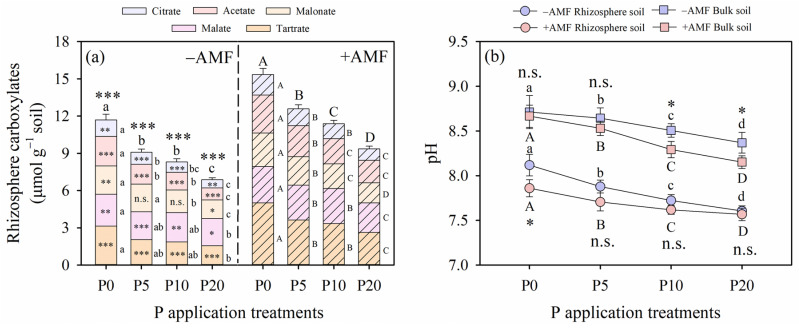
The rhizosphere carboxylates concentrations in alfalfa relative to rhizosphere soil mass (**a**) and pH value in the rhizosphere soil and bulk soil (**b**) under different arbuscular mycorrhizal fungi (AMF) inoculation and phosphorus (P) application treatments. Different lowercase letters and capital letters indicate significant differences among the P application rates under the −AMF and +AMF treatment, respectively. Levels of significance for differences between −AMF and +AMF treatments, which are indicated as: n.s. = not significant, * = *p* < 0.05, ** = *p* < 0.01, *** = *p* < 0.001. Vertical dashed lines denote the separation of −AMF and +AMF treatment. Data are reported as arithmetic mean ± standard error (n = 5).

As P application levels increased, rhizosphere carboxylates showed a gradual decline in all alfalfa plants across both AMF inoculation treatments (Figure 3a). In the +AMF treatment, the concentrations of citrate, acetate, malonate, malate, tartrate and the total rhizosphere carboxylates exhibited reductions of 44%, 41%, 40%, 18%, 48%, and 39%, respectively. In the −AMF treatment, the concentrations of these same carboxylates and total rhizosphere carboxylates exhibited reductions of 50%, 60%, 34%, 15%, 50%, and 42%, respectively.

The coefficients of variation (CV) for citrate, acetate, malonate, malate, tartrate, and the total rhizosphere carboxylates were 29% (−AMF) vs. 24% (+AMF), 34% (−AMF) vs. 23% (+AMF), 21% (−AMF) vs. 20% (+AMF), 12% (−AMF) vs. 11% (+AMF), 34% (−AMF) vs. 27% (+AMF), and 22% (−AMF) vs. 19% (+AMF), respectively. Notably, the total amount of rhizosphere carboxylates was significantly increased by AMF inoculation within the same P application level (*p* < 0.05). Both AMF inoculation and P application treatments had a significant effect on the rhizosphere carboxylates (*p* < 0.05; Table 3).

### 2.4. Soil pH, Available P Content, Alkaline Phosphatase Activity, Microbial Biomass P and Microbial Biomass C

**Figure 4:**
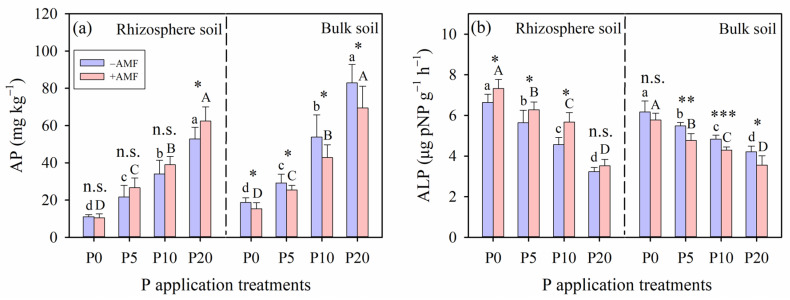
(**a**) Available phosphorus content (AP) and (**b**) alkaline phosphatase activity (ALP) in the rhizosphere soil and bulk soil under different arbuscular mycorrhizal fungi (AMF) inoculation and phosphorus (P) application treatments. Different lowercase letters and capital letters indicate significant differences among the P application rates under the −AMF and +AMF treatments, respectively. Levels of significance for differences between −AMF and +AMF treatments are indicated as: n.s. = not significant, * = *p* < 0.05, ** = *p* < 0.01, *** = *p* < 0.001. Vertical dashed lines denote the separation of rhizosphere soil and bulk soil. Data are reported as arithmetic mean ± standard error.

In both rhizosphere and bulk soils, AMF inoculation and P application treatments significantly influenced soil pH, available P content (AP, Olsen-P), alkaline phosphatase activity (ALP), microbial biomass P (MBP), and microbial biomass carbon (MBC) (*p* < 0.05; Table 3). As P application levels increased across all the AMF inoculation treatments, soil pH and ALP decreased, while AP increased in both the rhizosphere and bulk soils (Figure 3a and Figure 4). AMF inoculation did not significantly affect rhizosphere soil pH within the same P application level, except in the P0 treatment (Figure 3b). Conversely, bulk soil pH was significantly higher under the +AMF treatment compared to that under the −AMF treatment at the P10 and P20 application levels.

AMF inoculation enhanced AP in the rhizosphere soil, and this enhancement also had significant effects on the AP in the bulk soil (*p* < 0.05; Figure 4a). Notably, the content of AP in the rhizosphere soil was consistently lower than that in the bulk soil across all P application levels within the same AMF inoculation treatment. No significant differences in the AP between +AMF and −AMF treatments were observed at all P application levels, except for the P20 application level in rhizosphere soil (*p* > 0.05; Figure 4a). Alkaline phosphatase activity in the rhizosphere was significantly higher than that in the bulk soil (*p* < 0.05), except in the P20 treatment, where no significant difference was detected (*p* > 0.05; Figure 4b).

**Figure 5:**
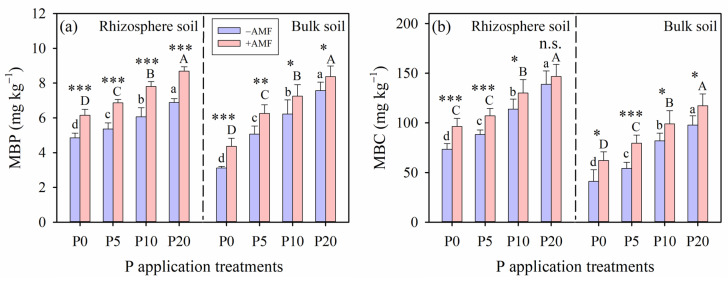
(**a**) Microbial biomass phosphorus (P) and (**b**) microbial biomass carbon (C) under different arbuscular mycorrhizal fungi (AMF) inoculation and phosphorus (P) application treatments in the rhizosphere soil and bulk soil, respectively. Different lowercase (for −AMF) and capital (for +AMF) letters indicate significant differences among P application rates. Levels of significance for differences between −AMF and +AMF treatments, which are indicated as: n.s. = not significant, * = *p* < 0.05, ** = *p* < 0.01, *** = *p* < 0.001. Vertical dashed lines denote the separation between rhizosphere and bulk soils. Data are reported as arithmetic mean ± standard error (n = 5).

Under both the +AMF and −AMF treatments, MBP and MBC significantly increased with higher P application levels in both the rhizosphere and bulk soils, ultimately peaking at the P20 treatment (*p* < 0.05; Figure 5). Within the same P application level, MBP under the +AMF treatment was significantly higher compared to that under the −AMF treatment both in the rhizosphere and bulk soils (*p* < 0.05; Figure 5a). AMF inoculation significantly influenced soil MBC, although this effect was not observed in the rhizosphere soil under the P20 treatment (*p* > 0.05; Figure 5b). Across both the AMF inoculation treatments, MBC was consistently higher in the rhizosphere soil than that in the bulk soil within the same P application level (*p* < 0.05; Figure 5b).

### 2.5. Correlation Analysis Between P-Utilization Efficiency and Rhizosphere Soil Variables, and Rhizosphere Carboxylates

**Figure 6:**
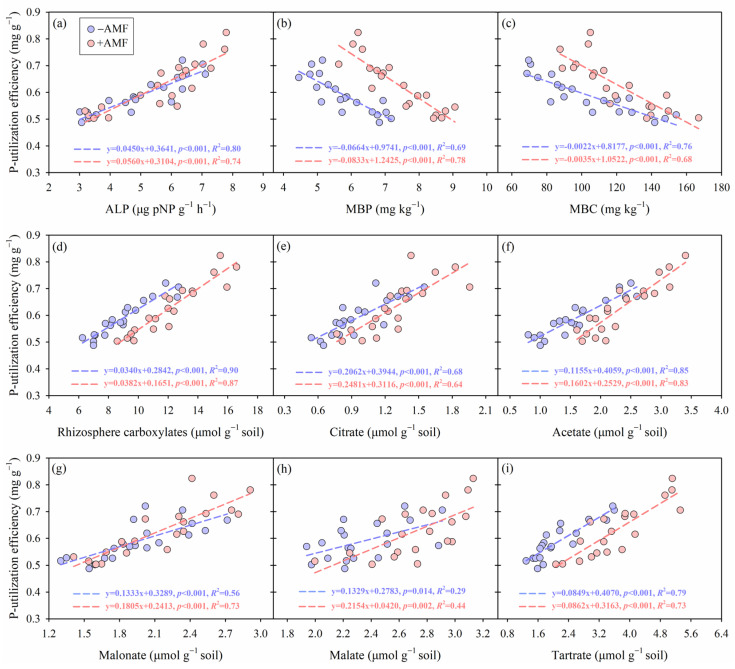
The relationships between phosphorus (P) utilization efficiency and (**a**) alkaline phosphatase activity, (**b**) microbial biomass phosphorus (P), (**c**) microbial biomass carbon (C), (**d**) total rhizosphere carboxylates, (**e**) citrate, (**f**) acetate, (**g**) malonate, (**h**) malate, and (**i**) tartrate content in alfalfa rhizosphere, respectively. The blue and red dotted lines represent the fitted standard major axis regression lines for the −AMF and +AMF treatments, respectively. Linear regression *R*^2^ values and significance levels (*p*-values) are provided for each relationship.

For both +AMF and −AMF treatments, P-utilization efficiency was significantly correlated with several rhizosphere soil parameters and rhizosphere carboxylates, including ALP, MBP, MBC, total concentration of rhizosphere carboxylates, as well as individual carboxylates such as citrate, acetate, malonate, malate, and tartrate (*p* < 0.05; Figure 6). Regression analysis revealed positive correlations across all linear models, except for MBP and MBC, which exhibited significant negative correlations with P-utilization efficiency (*p* < 0.05, Figure 6b,c). Additionally, the regression slopes differed between +AMF and −AMF treatments, indicating that AMF inoculation modified these relationships. Specifically, the slopes of the positive correlations were steeper under the +AMF treatments compared to the −AMF treatments, suggesting that AMF inoculation amplified the strength of these positive correlations, except for the negative correlations with MBP and MBC.

**Figure 7:**
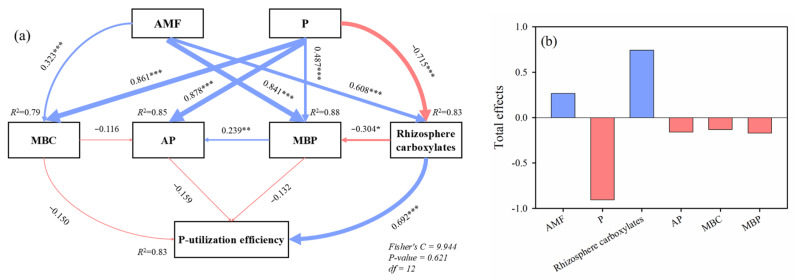
(**a**) Structural equation model (SEM) of phosphorus (P) utilization efficiency of alfalfa under different arbuscular mycorrhizal fungi (AMF) inoculation and P application treatments and (**b**) total effects of different influencing factors on alfalfa P-utilization efficiency derived from the SEM analysis. The red and blue arrows represent positive and negative flows of causality, respectively. * = *p* < 0.05, ** = *p* < 0.01, *** = *p* < 0.001. The thickness of the line represents the magnitude of influence. Numbers on the arrow indicate significant standardized path coefficients.

The SEM analysis elucidated the path relationships governing the P-utilization efficiency of alfalfa plants and its influencing factors under different AMF inoculation and P application treatments (Figure 7). Following adjustments based on the model modification indices, SEM demonstrated an appropriate fit with our data (χ^2^ = 9.944, *df* = 12, *p* = 0.621). The variation in P-utilization efficiency was influenced by both direct and indirect effects stemming from rhizosphere soil AP, MBP, MBC, and rhizosphere carboxylates (Figure 7a). The AMF colonization (0.266) and rhizosphere carboxylates (0.744) had positive total effects on the P-utilization efficiency of alfalfa, while P application (−0.907), soil AP (−0.159), MBP (−0.132), and MBC (−0.170) showed negative total effects on the P-utilization efficiency of alfalfa (Figure 7b). Thus, the strongest positive influence on P-utilization efficiency stemmed from rhizosphere carboxylates under different AMF inoculation and P application treatments.

## 3. Discussion

Phosphorus (P) deficiency is one of the primary nutrient limiting factors for alfalfa growth and productivity in the saline-alkali soil. P fertilizers, as an agronomic management practice, could temporarily alleviate low P stress in crops; however, large amounts of soluble fertilizer applications could also increase production costs and environmental pollution. In this experiment, we aimed to explore the potential mechanisms and related processes underlying the synergistic effects of arbuscular mycorrhizal fungi (AMF) inoculation with P application to enhance the efficiency of P acquisition and utilization in alfalfa under varying P application levels. The main finding of the present experiment was that AMF could improve soil environment conditions, microbial biomass and root functional traits—especially by increasing the carboxylates in root exudates, and improving the efficiencies of P acquisition and utilization in alfalfa under low-P supply conditions.

### 3.1. Effects of AMF Inoculation on Alfalfa Growth and AMF Status Under Different P Levels

Our results show that AMF inoculation significantly enhanced alfalfa biomass as well as shoot and root P concentrations across all P application levels, except in the highest P treatment (P20) (Table 2; Figure 1). This increase in biomass under AMF inoculation, particularly in the low (P0 and P5) and moderate (P10) P treatments, supports the hypothesis that AMF can improve plant growth under P-deficient conditions. These findings are consistent with previous studies [7,39], which reported that AMF colonization enhances plant biomass by improving P uptake, water use efficiency and overall nutrient acquisition via external hyphae [27,40]. Notably, the highest levels of mycorrhizal colonization rate, spore density, hyphal length and mycorrhizal contribution to P acquisition were observed under the P0 treatment. In contrast, these parameters declined markedly under the P20 treatment (Table 1), suggesting that high P availability reduces plant dependence on AMF. This decline in AMF development under high P supply aligns with Grman et al. (2012) [41] and Huo et al. (2022) [42], who reported that high P availability reduces plant-mycorrhizal associations, observing diminished AMF effectiveness in P-rich soils where plant’s dependence on mycorrhizal associations is reduced.

In this study, we observed a negative correlation between soil available P and AMF colonization rate, which decreased with increasing P supply (from P5 to P20). This is consistent with earlier findings [39,43]. Three mechanisms may explain the enhanced AMF colonization under low P supply. First, from a soil perspective, many studies have shown that when available P in soil is below 50 mg kg^−1^, AMF colonization increases [7,38,39,44]. In our study, available P concentrations in both rhizosphere and bulk soils remained below this threshold in the P0 and P5 treatments (Figure 4). Second, from the AMF perspective, mycorrhizal associations shift from mutualistic to parasitic when the net carbon cost to the host outweighs the benefit, a scenario more likely in P-sufficient soils [37,45]. Third, from the host plant perspective, plants adjust carbon allocation depending on nutrient availability [28,46]. AMF symbiosis requires substantial carbon investment from the host plant; thus, under P deficiency, plants may prioritize this carbon allocation to enhance nutrient uptake despite the metabolic cost [6,47]. Furthermore, our observation that the lowest AMF colonization rate and mycorrhizal contribution were detected at the highest P application levels (P20 treatment), which suggests that the relative contribution of AMF to P uptake may decline, as alfalfa could meet its P needs without the assistance of mycorrhizal fungi under conditions of high P availability. Together, these findings suggest that AMF contribution to P uptake is most effective under low-P conditions and diminishes when external P supply is sufficient to meet plant demand.

### 3.2. P-Acquisition Strategy of AMF Inoculation to Improve P-Use Efficiency in Alfalfa Under Low-P Supply Conditions

Our results demonstrate that AMF inoculation significantly improved both P acquisition efficiency and P utilization efficiency under the low (P0 and P5) to the moderate (P10) P supply conditions (Figure 2d,e); this trend supports previous findings that AMF inoculation is particularly advantageous in P-limited environments [7,25,28]. However, as P availability increased, the benefits conferred by AMF declined, suggesting that plants rely less on mycorrhizal associations when P is abundant, which further underscores a trade-off between plants’ demand for P acquisition and mycorrhizal colonization.

The enhancement of plant P acquisition is closely related to mycorrhizal parameters, particularly hyphal length (Table 1), which extends the nutrient absorption capacity of the root system [26,48]. In addition to the role of mycorrhizal parameters, in general, consistent with the findings of this study, compatible AMF-symbiotic plants rely on specialized “mycorrhizal phosphate uptake” (MPU) pathways, where mycorrhizal hyphae deliver P from distant soil regions [29]. In this study, the lower root-to-shoot ratio in AMF-inoculated alfalfa suggests that the AM hyphal network substituted for fine root proliferation, reducing the plant’s carbon cost for root development [49,50,51]. Moreover, the MPU pathway allows for faster and broader P transport than the direct pathway (DP), which depends on root hairs and epidermal cells [29,50]. The fact that the MPU pathway allows for faster and broader P transport, thereby enabling the plant to access immobile or poorly soluble P fractions enhances the plant’s ability to access immobile or poorly soluble P fractions, particularly under P-deficient conditions.

Rhizosphere carboxylate exudation is another important strategy for P acquisition [5,20]. Our results showed that AMF-inoculated plants consistently had higher rhizosphere carboxylate concentrations than non-inoculated plants (Figure 3a), indicating AMF’s role in promoting root exudation processes, which are critical for mobilizing phosphorus from less soluble P pools, particularly in the phosphorus-deficient soils [52,53]. These findings are consistent with research by Kadowaki et al. (2018) [54], which showed that AMF enhances root exudation of organic acids, thereby improving phosphorus solubilization and uptake. Moreover, a positive correlation between rhizosphere carboxylates concentration and P-utilization efficiency further supports the contribution of AMF to enhancing root exudation under low P supply (Figure 6 and Figure 7). Interestingly, we found that P levels led to a decline in rhizosphere carboxylate exudates in alfalfa (Figure 3a and Figure 7), suggesting a trade-off in carbon allocation between supporting AMF and producing exudates [6,52]. At higher P levels (P20), the plateau in P acquisition efficiency indicates that plants rely more on direct uptake and less on symbiotic or exudate-mediated strategies [13,23]. In addition to affecting root exudation, AMF inoculation slightly decreased rhizosphere pH under the P0 treatment (Figure 3b), potentially enhancing P solubility in saline-alkali soils [22,24,25,55]. While AMF had little effect on pH under higher P levels, it significantly increased Olsen-P content under low-P treatments (Figure 4), suggesting improved solubilization and mobilization of unavailable P fractions [25,56,57,58,59].

Rhizosphere Olsen-P concentrations reflect the dynamic equilibrium between plant P uptake and rhizosphere P solubilization processes, with AMF playing a pivotal mediating role in modulating this balance [16]. Our findings demonstrated that AMF inoculation significantly elevated rhizosphere Olsen-P concentrations of alfalfa relative to the non-inoculation control (Figure 4), which can be attributed to the synergistic effects of AMF-mediated plant P acquisition and enhanced rhizosphere P solubilization [7]. First, AMF inoculation significantly increased biomass and plant P accumulation in alfalfa, which indicates that the elevated P demand associated with AMF-plant symbiosis was partially offset by enhanced rhizosphere P availability [8]. Notably, AMF hyphae can extend beyond the rhizosphere P depletion zone to access otherwise unavailable P pools, which may alleviate direct competition for rhizosphere available P between plant roots and soil microorganisms [26]. This hyphal-mediated P acquisition further contributes to the maintenance of higher rhizosphere Olsen-P concentrations in inoculated treatments [25]. Second, our data revealed that AMF inoculation significantly increased rhizosphere carboxylate concentrations (Figure 3). Carboxylates are well-recognized for their ability to chelate Ca^2+^/Mg^2+^ in saline-alkaline soils and dissolve sparingly soluble inorganic P minerals (e.g., Ca_3_(PO_4_)_2_) into plant-available orthophosphate [32]. Moreover, AMF may indirectly enhance carboxylate secretion by regulating plant root exudation metabolism (e.g., upregulating genes involved in carboxylate biosynthesis) or modifying rhizosphere pH, thereby creating a more conducive microenvironment for P solubilization [16]. Collectively, these results indicate that AMF inoculation shifts the balance between plant P uptake and rhizosphere P solubilization toward enhanced P solubilization, while mitigating Olsen-P depletion through hyphal P acquisition. The findings regarding AMF’s effects on rhizosphere carboxylates concentration, pH and Olsen-P content support the hypothesis that AMF improves P availability through biological and biochemical modifications of the rhizosphere.

AMF also positively affected microbial activity [18,39]. This increase in microbial activity may also improve soil structure and function, supporting longer-term soil fertility and sustainability [60]. The research’s results reveal that microbial biomass phosphorus (P) and microbial biomass carbon (C) were significantly higher in AMF-inoculated soils under low-P conditions (Figure 5), consistent with earlier studies [18,39,61]. This increase in microbial biomass suggests that AMF inoculation enhances soil microbial activity beyond the rhizosphere, which may contribute to long-term nutrient cycling and soil fertility [47,57,60]. Moreover, alkaline phosphatase activity, an indicator of organic P mineralization, was significantly higher in AMF-treated rhizosphere soils, especially under low-P conditions [62]. This suggests that AMF stimulates enzymatic breakdown of organic P compounds, further increasing P availability for plant uptake [25,57,58]. These results highlight the broader role of AMF not only in enhancing direct P acquisition but also in improving rhizosphere biochemical activity and microbial-mediated nutrient cycling in both rhizosphere and bulk soils.

This study highlights the crucial role of AMF inoculation combined with P application in synergistically increasing carboxylate concentrations in root exudates and enhancing alfalfa’s P acquisition and utilization efficiency in saline-alkali soil. While this study provides valuable insights through a well-designed experiment and yielded promising results, we acknowledge certain limitations that merit recognition. First, it is well established that alfalfa roots can intrinsically secrete carboxylates independently of AMF hyphae [26]. However, no direct evidence was provided in the present study to confirm a causal link between carboxylate secretion and improved P acquisition efficiency; thus, we cannot definitively attribute the observed enhancements in P acquisition solely to carboxylate-mediated P solubilization. Other AMF-related mechanisms (e.g., hyphal P uptake beyond the rhizosphere depletion zone) may also contribute, and their relative importance remains unquantified herein. Second, pot experiments may alter AMF colonization dynamics and root morphological traits, which could indirectly influence alfalfa’s P acquisition and utilization efficiency. Third, this study focused on a single alfalfa variety inoculated with a single AMF species, thus limiting the direct generalizability of the findings to other alfalfa genotypes or ecosystems harboring naturally diverse AMF communities. Therefore, future research should disentangle the relative contributions of alfalfa root-derived (AMF-independent) carboxylates and AMF-mediated carboxylate secretion via specialized experimental approaches (e.g., hyphal exclusion compartments to separate hyphal and root effects, or carboxylate secretion inhibitors). Meanwhile, field experiments be conducted to explore the mechanisms by which native AMF inoculation, combined with P application, improves P acquisition and utilization efficiency in alfalfa under low-P supply conditions in the saline-alkali soils of the Yellow River Delta.

## 4. Materials and Methods

### 4.1. Plant and Soil Preparation

Alfalfa (*Medicago sativa* L. cv. ‘Zhongmu No.3’), cultivated by the Institute of Animal Sciences of Chinese Academy of Agricultural Sciences, was selected for this study. Experimental artificial pasture microcosms were established using saline-alkali soil, collected from the topsoil (0–25 cm depth) of a wasteland (37°54′ N, 117°57′ E) near Binzhou City, Shandong Province, China [63]. The detailed physicochemical properties of the saline-alkali soil are provided in Table 4. Soil particle size distribution (i.e., sand, silt, and clay fractions) was determined following the pipette method described by Zhong et al. (2025) [64]. Soil pH and electrical conductivity (EC) were measured using a pH meter (PHS-3C, Leici Instrument Co., Ltd., Shanghai, China) and a conductivity meter (DDS-307, Leici Instrument Co., Ltd., Shanghai, China), respectively. Prior to measurements, the pH meter was calibrated with three-point standard buffer solutions (pH 4.00, 6.86, and 9.18), while the conductivity meter was calibrated against a 1413 μS·cm^−1^ standard solution to ensure measurement accuracy [35]. Soil cation exchange capacity (CEC) was determined using the ammonium acetate (NH_4_OAc) method at pH 7.0 [65]. Soil total carbon (TC) and total nitrogen (TN) were co-determined via oxidative dry combustion at 950 °C with an elemental analyzer (Vario EL cube, Elementar Analysensysteme GmbH, Hanau, Germany) [66]. Soil organic matter (SOM) was determined via the potassium dichromate (K_2_Cr_2_O_7_) oxidation-spectrophotometric method, involving digestion with concentrated sulfuric acid (H_2_SO_4_) followed by spectrophotometric quantification at 590 nm [67]. Soil total potassium (TK) was determined via mixed acid digestion with hydrofluoric acid (HF) and perchloric acid (HClO_4_) (*v*/*v*, typically 4:1) to dissolve silicate-bound K, followed by quantification via a flame photometer (FP6400, Shimadzu Corporation, Kyoto, Japan) [68].

The soil was air-dried, sieved through a 2 mm mesh to remove large stones and plant roots, and sterilized twice at 121 °C for 2 h using high-pressure steam to eliminate indigenous arbuscular mycorrhizal fungi (AMF). In this study, the AMF inoculum used was the species of *Glomus mosseae*, which was reported as a native and dominant AMF in the saline-alkali soil from the Yellow River Delta [69]. The AMF inoculum species was provided by the Bank of Glomeromycota in China, and the fungal isolate was cultivated using *Zea mays* L. as the host species in pot cultures containing sterilized sand. After four months, the substrate from these cultures was collected, consisting of spores (~60 spores g^−1^), infected root fragments, hyphae, and sand.

### 4.2. Experimental Design

The experiment was arranged in a completely randomized block design and conducted for 120 days in a greenhouse at Qingdao Agricultural University, Qingdao City, Shandong Province, China. The plants were kept under controlled conditions in a greenhouse with 25/20 °C day/night temperature, and 65–70% relative humidity. The photoperiod was maintained at 12 h (from 7:00 to 19:00), supplemented with high-pressure sodium lamps delivering a photosynthetic photon flux density (PPFD) of 400 μmol m^−2^ s^−1^ at a height of 2 m above the pot level. Two AMF inoculation treatments were applied: −AMF (without AMF inoculation) and +AMF (with AMF inoculation). Additionally, four phosphorus (P) application levels were tested: P0 (0 mg kg^−1^), P5 (5 mg kg^−1^), P10 (10 mg kg^−1^), and P20 (20 mg kg^−1^). Each pot was filled with 10 kg of sterilized soil. For the +AMF treatment, 200 g of AMF inoculum was added. For the −AMF treatment, an equal amount of AMF inoculum sterilized at 121 °C for 2 h was added to maintain consistent soil properties between treatments. Following the methodology outlined by Lendzemo et al. (2007) [70], unsterilized test soil was prepared by mixing and stirring, after which the soil suspension was filtered through a 20 μm sieve at a 2:1 water-to-soil ratio to eliminate large microorganisms, including AMF. The resulting filtrate was subsequently applied to the pots to reintroduce essential microbial communities and minimize differences in other microbial communities to the previously sterilized soil. Monopotassium phosphate (KH_2_PO_4_, analytically pure) was applied at 0 mg kg^−1^, 5 mg kg^−1^, 10 mg kg^−1^, and 20 mg kg^−1^ for the P0, P5, P10, and P20 treatments, respectively. NH_4_NO_3_ (analytically pure, 150 mg kg^−1^) and KCl (analytically pure, 100 mg kg^−1^) were applied to all treatments to ensure that plant growth was not limited by nutrients other than P. Each combination of AMF inoculation and P application treatments had five biological replicates, resulting in a total of 40 pots.

The seeds of *M. sativa* were surface disinfected with 30% (*v*/*v*) hydrogen peroxide for 5 min and rinsed five times with deionized (DI) water before germinating on moist filter paper at 20 °C for 48 h. Twenty seedlings were transplanted into each pot, and the seedlings were thinned to fifteen individuals per pot. To ensure that alfalfa growth was not limited by soil water deficit or affected by microenvironmental variation, the pots were watered to approximately 70% of field capacity, and the plants were periodically rearranged to maintain a repositioned distribution throughout the experiment [71]. The bottom of each pot was fitted with impermeable trays, and the leachate was collected periodically and returned to the pots to maintain the saline-alkaline condition in the soil.

### 4.3. Sample Collection

After 120 days of treatment, shoot material was carefully harvested by clipping at ground level from each experimental pot. Root systems were gently shaken to remove loosely adhering soil, with the remaining soil attached to the roots defined as rhizosphere soil [58]. Rhizosphere soil and bulk soil (non-rhizosphere soil, not directly associated with roots) samples were collected from each treatment after alfalfa harvesting and divided into two portions: one portion was air-dried for analysis of soil pH and available P content (AP), and the other was stored at −80 °C for the determination of alkaline phosphatase activity (ALP), microbial biomass phosphorus (MBP) and microbial biomass carbon (MBC).

For the determination of rhizosphere carboxylate exudates of alfalfa, approximately 1.5 g of fresh roots with attached rhizosphere soil were transferred to a beaker containing 20 mL of 0.2 mM CaCl_2_ to maintain cell integrity, and gently shaken to remove the rhizosphere soil from the roots. After collecting the rhizosphere carboxylates, the alfalfa roots were carefully washed with DI water, placed in individual zip-lock plastic bags, and stored at 4 °C for the evaluation of AMF characteristics, including AMF colonization rate, spore density and hyphal length.

### 4.4. Root Characteristics and Rhizosphere Mycorrhizal Status Measurements

After harvest, five plants from each treatment were randomly selected, and the main root characteristics were measured. Total root length, root diameter, and root surface area were digitized using an LA-S scanner (LA-S, Wanseng, Hangzhou, China) and analyzed with WinRhizo software (Version Pro 2019, Regent Instruments Inc., Quebec, QC, Canada). Specific root length was calculated as the ratio of root length to root dry mass. Fresh root subsamples were randomly taken to evaluate the percentage of root length colonized by AMF. For each treatment pot, roots were stained with 0.05% (*w*/*v*) Trypan blue, cut into approximately 1 cm (in length) fragments, and mounted on three slides for observation with the aid of a light microscope (Primo Star HD, Carl Zeiss AG, Jena, Germany) [37]. The percentage of root length colonized by AMF was determined using a modified line-intersection method with 100 intersections [72]. AMF spores were extracted from the air-dried rhizosphere soil samples using sucrose centrifugation and wet sieving techniques, and then manually quantified under a stereoscopic microscope (Stemi 305, Carl Zeiss AG, Oberkochen, Germany) [73]. External hyphal length in the air-dried rhizosphere soil was measured using the method of Bethlenfalvay and Ames (1987) [74].

### 4.5. Plant Biomass and P Concentration Measurements

Shoot and root samples were washed and heated at 105 °C for 30 min, then oven-dried at 65 °C to a constant weight and weighed separately to obtain the dry biomass. The root/shoot ratio was calculated as root biomass divided by shoot biomass. Total biomass was calculated as shoot and root biomass [75].

The oven-dried shoot and root samples were ground using a ball mill (MM400, Retsch, Haan, Germany), and subsamples were used to determine the P concentration in shoot and root, respectively. For each treatment, approximately 0.5 g of sample was digested with a mixture of nitric acid (HNO_3_) and perchloric acid (HClO_4_) at a ratio of 4:1 (*v*/*v*). P concentration was then determined using the molybdenum blue method after digestion [76].

The content of P in shoot and root was calculated as

$$P\;content\;in\;X={Biomass}_{X}\times P\;{concentration}_{X}$$

where X represents shoot and root, respectively, Biomass represents dry mass, and P concentration represents P content per unit mass.

The mycorrhizal P absorption contribution rate was calculated using the following equation [39]:

$$Mycorrhizal\;contribution=\frac{{Plant\;P}_{+AMF}{-Plant\;P}_{-AMF}}{{Plant\;P}_{+AMF}}\times 100\%$$

where Plant P_−AMF_ and Plant P_+AMF_ represents the total plant P content in without AMF inoculation (−AMF) and with AMF inoculation (+AMF) treatments under different P application levels, respectively.

The P-utilization efficiency was calculated according to the following formula:

$$P-utilization\;efficiency=\frac{Plant\;dry\;mass}{Plant\;P\;content}$$

where Plant dry mass represents the total biomass in each treatment and Plant P content was calculated as the summation of shoot P content and root P content.

The P-absorption efficiency was calculated as:

$$P-absorption\;efficiency=\frac{P_{X}-P_{0}}{P_{A}}$$

where P_0_ represents the total plant P content in the P_0_ application treatment, P_x_ represents the total P content in alfalfa seedlings under P5, P10 or P20 application treatment, respectively; P_A_ represents the total amount of P application in each experimental pot.

### 4.6. Rhizosphere Carboxylates Measurements

Rhizosphere carboxylates were extracted for analysis using High-Performance Liquid Chromatography (HPLC) following the method outlined by He et al. (2017) [46]. A subsample of 1 mL of the rhizosphere extract was filtered through a 0.22-μm syringe filter into an HPLC vial, acidified with a drop of concentrated phosphoric acid, and stored at −20 °C until analysis. Carboxylates in the extract were quantified using an HPLC system (Ultimate 3000, Thermo Fisher Scientific, Waltham, MA, USA), equipped with detector and reverse phase column (250 mm × 4.6 mm, 5 μm particle size). The mobile phase was 0.02 mol L^−1^ KH_2_PO_4_ buffer mixed with HPLC-grade methanol (85:15, *v*/*v*), at a flow rate of 1.0 mL min^−1^. The flow rate was set at 1.0 mL min^−1^, and the injection volume was 20 μL. To identify carboxylates such as citrate, acetate, malonate, malate, and tartrate, working standards of citric acid, acetic acid, malonic acid, malic acid, and tartaric acid were employed, with detection set at 210 nm, based on the approach of Cawthray (2003) [77]. The retention times of observed peaks were compared with those of the standards, and the identified organic acids were quantified using the calibration curves. All standards and dilutions were made with ion-exchanged water from a Milli-Q system. After carboxylate extraction, roots were rinsed thoroughly, oven-dried at 65 °C to constant weight, and their dry mass was recorded. The concentrations of rhizosphere carboxylate were expressed on a rhizosphere soil mass basis.

### 4.7. Soil pH, Available P Content and Alkaline Phosphatase Activity, Microbial Biomass P and Microbial Biomass C Measurements

Soil pH was measured using a pH meter (FE28-Standard, Mettler-Toledo Instruments Shanghai Co., Ltd., Shanghai, China) in a 1:2.5 (*w*/*v*) soil-to-water suspension [78]. Available P (Olsen-P) in the soil was extracted with 0.5 M NaHCO_3_ following the Olsen method [79] and measured using the protocol of Watanabe and Olsen (1965) [80]. Alkaline phosphatase (EC 3.1.3.1) activity was measured with assay kits (SAKP-1-W, Suzhou Comin Biotechnology Co., Suzhou, China). Approximately 5.0 g of fresh soil was incubated with toluene and suspended in sodium carbonate-sodium bicarbonate buffer containing p-nitrophenyl phosphate (pNP) at 37 °C for 24 h. Soil alkaline phosphatase activity was determined based on the absorbance of the released p-nitrophenol [62].

Soil microbial biomass P (MBP) and carbon (MBC) were determined using the chloroform fumigation-extraction method, performed in duplicate on 20 g subsamples using 80 mL of 0.5 M K_2_SO_4_ solution. Soil microbial biomass P and C were calculated according to Vance, Brookes and Jenkinson (1987) [61] and Jöergensen (1996) [81], respectively. Additionally, AMF hyphae are inactivated by the chloroform treatment, meaning that soil microbial biomass P and C measurements also include the P and C content of AMF [82].

### 4.8. Statistical Analysis

The data were evaluated for normal distribution using the Shapiro–Wilk test and for homoscedasticity using Levene’s test. A one-way analysis of variance (ANOVA) followed by Tukey’s multiple comparison test was performed to evaluate the effects of P application within each AMF inoculation treatment (−AMF or +AMF). Independent-samples *t*-tests were conducted to detect differences between AMF inoculation treatments for all indices. A two-way ANOVA was used to assess the main effects of AMF inoculation treatments (AMF) and P application (P), as well as their interaction effects (AMF × P). Additionally, linear regression analysis was performed to evaluate the dependence of P-utilization efficiency on rhizosphere soil parameters and rhizosphere carboxylates. All data analyses were conducted using SPSS software version 22 (SPSS Inc., Chicago, IL, USA), and the assumptions of residual normality were met for all analyses (Kolmogorov–Smirnov test). All figures were generated using SigmaPlot version 12.5 (Systat Software Inc., San Jose, CA, USA). A structural equation model (SEM) was established using the AMOS software version 26 (SPSS Inc., Chicago, IL, USA) to identify the major pathways of the influence of predictor variables on the P-utilization efficiency of alfalfa plants under different AMF inoculation and P application treatments. Furthermore, we investigated the standard total effects of different influencing factors on the P-utilization efficiency of alfalfa plants using SEM.

## 5. Conclusions

Overall, our results demonstrate that AMF colonization and P application synergistically enhanced the biomass accumulation and P acquisition of alfalfa (*Medicago sativa* L.) seedlings under low P availability in saline-alkali soils. Notably, AMF significantly enhanced P-utilization efficiency by increasing rhizosphere carboxylate exudation, particularly under a low P application level of 5 mg kg^−1^. In conclusion, the synergy between AMF colonization and low P application enhanced P acquisition and utilization efficiency in alfalfa via altering rhizosphere biochemical processes in saline-alkali soil. Future research needs to be conducted with more varieties of *M. sativa* in long-term field trials to understand the roles of root system architecture, rhizosphere physio-chemistry and beneficial microbes in improving alfalfa growth and nutrient absorption under low soil P availability in saline-alkali environments.

## Figures and Tables

**Table 1:** Effects of the different arbuscular mycorrhizal fungi inoculation (−AMF: without AMF inoculation and +AMF: with AMF inoculation) and phosphorus application (P0: 0 mg kg^−1^, P5: 5 mg kg^−1^, P10: 10 mg kg^−1^, and P20: 20 mg kg^−1^) treatments on the AMF colonization rate, spore density, hyphal length and mycorrhizal contribution to P acquisition. Different capital letters within each line indicate significant differences (*p* < 0.05) among the P application rates under the +AMF treatment. ** = *p* < 0.01, *** = *p* < 0.001. Data are reported as arithmetic mean ± standard error (n = 5).

Treatment		AMF Colonization Rate (%)		Spore Density (No. g^−1^)		Hyphal Length (m g^−1^)		Mycorrhizal Contribution (%)	
P0	−AMF	-		-		-		21.17 ± 4.02 AB	
	+AMF	60.36 ± 3.78 B		40.32 ± 2.34 B		2.52 ± 0.21 B			
P5	−AMF	-		-		-		23.58 ± 2.51 A	
	+AMF	69.62 ± 4.52 A		52.75 ± 4.84 A		3.01 ± 0.19 A			
P10	−AMF	-		-		-		18.37 ± 3.30 B	
	+AMF	62.42 ± 7.66 AB		48.89 ± 3.46 A		2.62 ± 0.17 B			
P20	−AMF	-		-		-		8.37 ± 4.57 C	
	+AMF	52.34 ± 5.38 C		35.07 ± 2.85 C		1.85 ± 0.15 C			
Significance									
		*F*	*p*	*F*	*p*	*F*	*p*	*F*	*p*
AMF		-	-	-	-	-	-	-	-
P		8.26	0.002 **	26.11	<0.001 ***	35.77	<0.001 ***	16.46	<0.001 ***
AMF × P		-	-	-	-	-	-	-	-

**Table 2:** Effects of the different arbuscular mycorrhizal fungi inoculation (−AMF: without AMF inoculation and +AMF: with AMF inoculation) and phosphorus application (P0: 0 mg kg^−1^, P5: 5 mg kg^−1^, P10: 10 mg kg^−1^, and P20: 20 mg kg^−1^) treatments on the total root length, root diameter, root surface area, specific root length, root/shoot ratio, total biomass of the alfalfa. Different lowercase and capital letters within each line indicate significant differences (*p* < 0.05) among the P application rates under the −AMF and +AMF treatments, respectively. Results of two-way analysis of variance (F-value and *p*-value) on the effects of AMF inoculation (AMF), P application (P) and their interactions (AMF × P) on the root traits and biomass accumulation of alfalfa. Levels of significance represent significant differences, which are indicated as: n.s. = not significant, * = *p* < 0.05, ** = *p* < 0.01, *** = *p* < 0.001. Data are reported as arithmetic mean ± standard error (n = 5).

Treatment		Total Root Length (m)		Root Diameter (mm)		Root Surface Area (cm^2^)		Specific Root Length (m g^−1^)		Root/Shoot Ratio (None)		Total Biomass (g Pot^−1^)	
P0	−AMF	11.82 ± 0.88 d **		1.95 ± 0.13 d ^n.s.^		118.22 ± 17.01 d *		4.83 ± 0.54 a ^n.s.^		0.41 ± 0.08 a ^n.s.^		8.62 ± 0.99 d **	
	+AMF	13.54 ± 0.69 C		1.93 ± 0.05 D		147.34 ± 18.53 D		4.72 ± 0.38 A		0.38 ± 0.05 A		10.48 ± 0.51 D	
P5	−AMF	13.78 ± 0.57 c **		2.16 ± 0.16 c ^n.s.^		152.84 ± 11.50 c *		4.66 ± 0.43 ab ^n.s.^		0.37 ± 0.02 ab ^n.s.^		11.03 ± 0.86 c ***	
	+AMF	14.90 ± 0.98 B		2.13 ± 0.09 C		176.76 ± 16.31 C		4.39 ± 0.54 AB		0.36 ± 0.05 B		13.04 ± 0.39 C	
P10	−AMF	15.71 ± 0.31 b *		2.67 ± 0.10 b *		225.57 ± 20.01 b ^n.s.^		4.54 ± 0.24 b ^n.s.^		0.34 ± 0.05 ab ^n.s.^		13.71 ± 1.20 b **	
	+AMF	16.62 ± 0.56 A		2.56 ± 0.09 B		247.22 ± 29.77 B		4.35 ± 0.35 BC		0.31 ± 0.02 C		16.20 ± 0.82 B	
P20	−AMF	17.56 ± 0.80 a ^n.s.^		3.40 ± 0.19 a *		318.56±32.84 a ^n.s.^		4.49 ± 0.55 c ***		0.32 ± 0.04 b ^n.s.^		16.57 ± 1.31 a *	
	+AMF	16.86 ± 0.56 A		3.14 ± 0.14 A		307.42 ± 18.65 A		4.14 ± 0.15 C		0.30 ± 0.02 C		17.84 ± 1.65 A	
Significance		*F*	*p*	*F*	*p*	*F*	*p*	*F*	*p*	*F*	*p*	*F*	*p*
AMF		12.14	0.001 ***	7.71	0.009 **	5.39	0.027 *	9.50	0.004 **	2.45	0.127 ^n.s.^	33.46	<0.001 ***
P		84.53	<0.001 ***	227.19	<0.001 ***	137.45	<0.001 ***	6.98	0.001 ***	6.91	0.001 ***	103.05	<0.001 ***
AMF × P		5.64	0.003 **	2.03	0.129 ^n.s.^	1.79	0.170 ^n.s.^	0.47	0.703 ^n.s.^	0.07	0.973 ^n.s.^	0.584	0.630 ^n.s.^

**Table 3:** Statistical level of significance (*F*-value and *p*-value) of the two-way analysis of variance on the effects of arbuscular mycorrhizal fungi (AMF) inoculation, phosphorus (P) application and their interactions (AMF × P) on shoot and root biomass, shoot and root P content, plant total P content, P-utilization and P-acquisition efficiency of alfalfa, rhizosphere carboxylates contents (citrate, acetate, malonate, malate, tartrate and total amount) in alfalfa rhizosphere and pH value, available P content (AP), alkaline phosphatase activity (ALP), microbial biomass P (MBP) and microbial biomass carbon (MBC) in rhizosphere soil and bulk soils, respectively. n.s. = not significant, * = *p* < 0.05, ** = *p* < 0.01, *** = *p* < 0.001. Data are reported as arithmetic mean ± standard error (n = 5).

	Arbuscular Mycorrhizal Fungi (AMF)		Phosphorus (P)		AMF × P	
	*F*	*p*	*F*	*p*	*F*	*p*
Shoot biomass	25.02	<0.001 ***	77.54	<0.001 ***	0.42	0.738 ^n.s.^
Root biomass	14.43	0.001 **	43.41	<0.001 ***	0.66	0.582 ^n.s.^
Shoot P content	9.42	0.004 ***	60.76	<0.001 ***	1.05	0.386 ^n.s.^
Root P content	16.75	<0.001 ***	82.50	<0.001 ***	1.76	0.175 ^n.s.^
Plant P content	55.64	<0.001 ***	226.37	<0.001 ***	1.00	0.405 ^n.s.^
P-utilization efficiency	20.79	<0.001 ***	92.87	<0.001 ***	3.21	0.036 *
P-acquisition efficiency	10.89	0.003 **	20.47	<0.001 ***	2.00	0.157 ^n.s.^
Citrate	51.35	<0.001 ***	38.50	<0.001 ***	0.42	0.742 ^n.s.^
Acetate	145.34	<0.001 ***	82.28	<0.001 ***	1.59	0.313 ^n.s.^
Malonate	8.22	0.007 **	36.19	<0.001 ***	1.30	0.290 ^n.s.^
Malate	25.47	<0.001 ***	6.72	0.002 **	1.05	0.406 ^n.s.^
Tartrate	114.03	<0.001 ***	35.54	<0.001 ***	1.43	0.251 ^n.s.^
Rhizosphere carboxylates	191.21	<0.001 ***	96.48	<0.001 ***	1.28	0.300 ^n.s.^
Rhizosphere soil						
pH	6.09	0.019 *	9.08	<0.001 ***	0.66	0.583 ^n.s.^
AP	7.34	0.011 *	130.56	<0.001 ***	1.43	0.253 ^n.s.^
ALP	27.06	<0.001 ***	134.93	<0.001 ***	1.67	0.193 ^n.s.^
MBP	247.04	<0.001 ***	96.84	<0.001 ***	1.33	0.281 ^n.s.^
MBC	27.55	<0.001 ***	67.02	<0.001 ***	1.03	0.393 ^n.s.^
Bulk soil						
pH	4.14	0.050 *	6.79	0.001 **	0.35	0.790 ^n.s.^
AP	10.69	0.003 **	117.06	<0.001 ***	1.11	0.359 ^n.s.^
ALP	29.77	<0.001 ***	70.43	<0.001 ***	0.47	0.709 ^n.s.^
MBP	38.38	<0.001 ***	107.81	<0.001 ***	0.33	0.805 ^n.s.^
MBC	44.52	<0.001 ***	62.86	<0.001 ***	0.31	0.821 ^n.s.^

**Table 4:** Selected physicochemical properties of saline-alkali soil in the Yellow River Delta. Values are the arithmetic mean of five replicates (n = 5).

Factor	Soil
Clay (<0.002 mm), %	4
Silt (0.05–0.002 mm), %	70
Sand (2–0.05 mm), %	26
pH	8.97
EC, μS cm^−1^	1044.68
CEC, cmol + kg^−1^	23.05
Total C, g kg^−1^	18.38
Total N, mg kg^−1^	251.60
Total K, mg kg^−1^	19.38
Total P, mg kg^−1^	547.76
Available P, mg kg^−1^	3.76
Organic matter, g kg^−1^	5.19

## Data Availability

The data that support the findings of this study are available from the corresponding author upon reasonable request. The data generated in this study are not publicly available due to privacy constraints.
